# Levels of *P*-element-induced hybrid dysgenesis in *Drosophila simulans* are uncorrelated with levels of *P*-element piRNAs

**DOI:** 10.1093/g3journal/jkac324

**Published:** 2022-12-08

**Authors:** Olga Paulouskaya, Valèria Romero-Soriano, Claudia Ramirez-Lanzas, Tom A R Price, Andrea J Betancourt

**Affiliations:** Department of Evolution, Ecology and Behaviour, University of Liverpool, L69 7ZB Liverpool, UK; Institute of Biology Leiden, Leiden University, PO Box 9505, 2300 RA, Leiden, The Netherlands; Department of Evolution, Ecology and Behaviour, University of Liverpool, L69 7ZB Liverpool, UK; Institut für Populationsgenetik, Vetmeduni Vienna, A-1210 Vienna, Austria; Department of Evolution, Ecology and Behaviour, University of Liverpool, L69 7ZB Liverpool, UK; Department of Evolution, Ecology and Behaviour, University of Liverpool, L69 7ZB Liverpool, UK

**Keywords:** transposable elements, drosophila, piRNA

## Abstract

Transposable elements (TEs) are genomic parasites that proliferate within host genomes, and which can also invade new species. The *P*-element, a DNA-based TE, recently invaded two *Drosophila* species: *Drosophila melanogaster* in the 20th century, and *D. simulans* in the 21st. In both species, lines collected before the invasion are susceptible to “hybrid dysgenesis”, a syndrome of abnormal phenotypes apparently due to *P*-element-inflicted DNA damage. In *D. melanogaster*, lines collected after the invasion have evolved a maternally acting mechanism that suppresses hybrid dysgenesis, with extensive work showing that PIWI-interacting small RNAs (piRNAs) are a key factor in this suppression. Most of these studies use lines collected many generations after the initial *P*-element invasion. Here, we study *D. simulans* collected early, as well as late in the *P*-element invasion of this species. Like *D. melanogaster*, *D. simulans* from late in the invasion show strong resistance to hybrid dysgenesis and abundant *P*-element-derived piRNAs. Lines collected early in the invasion, however, show substantial variation in how much they suffer from hybrid dysgenesis, with some lines highly resistant. Surprisingly, although, these resistant lines do not show high levels of cognate maternal *P*-element piRNAs; in these lines, it may be that other mechanisms suppress hybrid dysgenesis.

## Introduction

Selfish genetic elements are parasitic genes that persist due to mechanisms that promote their own transmission, regardless of any deleterious effect on the host (Doolittle and Sapienza 1980; Orgel and Crick 1980; Werren *et al.* 1988; Burt and Trivers 2006). The most taxonomically widespread example of selfish genetic elements is transposable elements [TEs; reviewed in Wicker *et al.* (2007)]. TEs promote their own transmission by increasing their copy number within genomes by copying themselves from one location into another, or “transposing”. TEs are found in nearly all eukaryotic species investigated to date [reviewed in Gregory (2005)] and comprise large proportions of eukaryotic genomes [e.g. 95% of the maize genome (Kronmiller and Wise 2008)]. In addition to proliferating within genomes, TEs invade new species (reviewed in Schaack *et al.* 2010); in fact, as TEs are mostly deleterious, these invasions into naïve genomes may be key to their long-term persistence (Lohe *et al.* 1995; Blumenstiel 2011).

The best-known example of a TE invasion is that of the *P*-element in *D. melanogaster* in the 20th century. The *P*-element was initially discovered through crossing wild flies collected post-invasion with laboratory stocks maintained for decades prior to the invasion by fly geneticists. Specifically, in crosses where males carry *P*-elements and females lack them, the offspring can suffer from abnormal phenotypes, including high mutation rates, chromosomal rearrangements, atypically small gonads, recombination in males (abnormal for *Drosophila*) and sterility, collectively called hybrid dysgenesis (HD) (Hiraizumi 1971; Kidwell *et al.* 1977; Bingham *et al.* 1982; Kidwell 1983; Ghanim *et al.* 2020). The cause of HD appears to be unregulated *P*-element transposase, so that its endonuclease activity results in double-stranded DNA breaks ultimately triggering apoptosis of the developing germ cells (Tasnim and Kelleher 2017; Dorogova *et al.* 2017).

Due to these and other costs imposed by uncontrolled transposition, hosts have evolved several ways to suppress TEs. In the germline of *Drosophila* and other animals, TEs are thought to be regulated mainly by PIWI-interacting small RNAs (piRNAs)—piRNAs are encoded by sequences homologous to TEs, with specific TEs regulated by their cognate piRNAs [Brennecke *et al.* 2007; reviewed in Czech *et al.* (2018)]. In *Drosophila*, piRNAs are encoded by TE sequences concentrated into discrete clusters, expressed in ovaries, and then loaded into the egg by the female parent. *P*-element-induced HD is thought to occur when P-element cognate piRNAs are absent from the ovaries and eggs of the female parent (Brennecke *et al.* 2007).

To date, work on mechanisms of TE suppression has focused on TEs present in the host species for many generations. In *D. simulans*, the *P*-element became common only in the 2000s (Kofler *et al.* 2015; Hill *et al.* 2016), offering an opportunity to study the evolution of TE suppression early in an invasion. Here, we examine lines collected early in the *D. simulans* invasion for their ability to suppress *P*-element-induced HD. We specifically focus on gonadal HD in females, which in *D. simulans* results in ovaries that are absent or morphologically abnormal, and in the sterility of the affected females (Hill *et al.* 2016). The lines show substantial variation in their ability to suppress *P*-element-induced HD. Surprisingly, we were unable to find any association between maternal suppression of HD and maternal piRNA production for the *P*-element, suggesting that, in these lines, HD suppression, or tolerance of its effects, does not solely depend on expression levels of piRNAs.

## Materials and methods

### Fly lines

The *D. simulans* isofemale lines used in this study were collected from Georgia, USA, in 2009 (in Athens by P. Haddrill and in Morben by A. Paaby) or Croatia in 2014 (by A. Jakšić); the Croatia line *Cro18* was used as a P-type in our HD assays.

### HD assays

We assayed 28 isofemale lines for gonadal HD, as in Hill *et al*. (2016). Briefly, we crossed five virgin females to five tester males from the Cro18 P-type line at 29°C—a temperature at which gonadal dysgenesis is induced in both *D. melanogaster* and *D. simulans* (Kidwell *et al.* 1977; Hill *et al.* 2016; Ghanim *et al.* 2020) and allowed flies to lay eggs for a total of 8 days. We dissected 3–4 day-old F1 females from each cross and recorded the presence or absence of two well-formed ovaries. Females lacking two normal ovaries were considered to be dysgenic. We compared the proportions of dysgenic and normal females between reciprocal crosses with a Fisher's exact test.

### *P*-element screening

To check for the presence of full-length *P*-element copies in the genome of 12 chosen isofemale lines, we extracted DNA from 30 flies using the Qiagen DNeasy Blood & Tissue Kit and amplified each of the *P*-element exons separately using PCR. Primer sequences and PCR conditions are as in [10] (Supplementary Table 1).

### Quantitative PCR

We performed qPCR assay to estimate copy numbers of TEs (*P*-element and *hobo*) and to measure expression levels of the *P*-element. To estimate copy numbers, we extracted DNA from each line in three biological replicates (5–10 females per replicate) using the Qiagen DNeasy Blood & Tissue Kit. We used qPCR primers specific to the TE of interest and *rp49* as a reference gene for relative quantification (Livak and Schmittgen 2001). To investigate the expression and splicing efficiency of the *P*-element, we extracted total RNA from the dissected ovaries of 3–4 days-old flies using the NucleoSpin RNA kit (Macherey Nagel), and reverse-transcribed it with Roche's Transcriptor First cDNA synthesis kit. We then performed qPCR using two sets of primers for the *P*-element—one specific to exon 2 (to measure overall mRNA levels) and one spanning a splice boundary at intron 3 (to measure levels of spliced *P*-element mRNA), with *rp49* as the reference gene as before. We Box-Cox transformed the expression data (using R package MASS, Venables and Ripley 2002) and analyzed factors affecting levels of spliced *P*-element mRNA and the splicing efficiency (the ratio of spliced to total *P*-element mRNA) using an ANOVA (see results for specifics). For all quantitative PCR (qPCR), we used a Roche LightCycler 480 Instrument and KAPA SYBR FAST Universal Kit. Primer sequences and qPCR conditions are listed in Supplementary Table 1.

### Small RNA sequencing

We dissected ovaries from ten 3–4-day-old females, homogenized them in Trizol (Invitrogen) and froze the lysate in liquid nitrogen. We extracted total RNA using 5PRIME heavy Phase Lock Gel tubes, measured RNA concentration using Nanodrop, and assessed the quality of the samples on a denaturing agarose gel. Final quantification was performed using Agilent Bioanalyser. Library preparation and sequencing were performed by Fasteris (including a *Drosophila*-specific 2S RNA depletion step), for two biological replicates per line, on an Illumina HiSeq 4000 1 × 50 lane.

### Bioinformatic analyses

First, we removed 3′-adapters from the raw sequencing reads using Cutadapt software v1.10 (Martin 2011) and discarded reads shorter than 5 bp post-trimming. We mapped the remaining reads to a database of *Drosophila* TEs (annotation v. 6.42, available from http://www.flybase.org) twice, allowing first for three and then for six mismatches (-i 2 -l 40 -M 1 and -n 3 or -n 6) using *bwa* aln v 0.7.13 (Li and Durbin 2010). We then removed reads mapped with insertions and deletions with a custom bash script. The remaining reads were then mapped to *D. simulans* miRNAs database from FlyBase (dsim_r2.02_FB2017_04), using *bwa* aln (1 mismatch). Only reads that did not align to miRNAs were kept for further analyses. To test for differential expression, we used voom, implemented in the R Bioconductor package (Law *et al.* 2014). Voom estimates the mean-variance relationship in the data and uses it to compute weights for each gene (TE family in this case) and normalize the data, to allow tests for differential expression using standard log-linear models. We tested for a “ping-pong” signature, the presence of sense-antisense read pairs overlapping by 10 nucleotide characteristic of ping-pong processed piRNAs, using either a custom python script or the signature.py pipeline (Antoniewski 2014).

## Results

### Genetic variation in tolerance to *P*-element-induced HD

We performed an initial screen to quantify levels of HD in 28 isofemale lines collected from Georgia in Eastern North America in 2009 when the *P*-element invasion of *D. simulans* was in an early phase. Most of these lines lacked full-length, potentially active *P*-elements, but many contain partial copies (Hill *et al.* 2016). We confirmed this result by attempting to amplify each of the four exons of the *P*-element: we were able to amplify all four exons in seven, some exons in 19, and none in two of 28 lines (Supplementary Table 2).

**Fig. 1.**
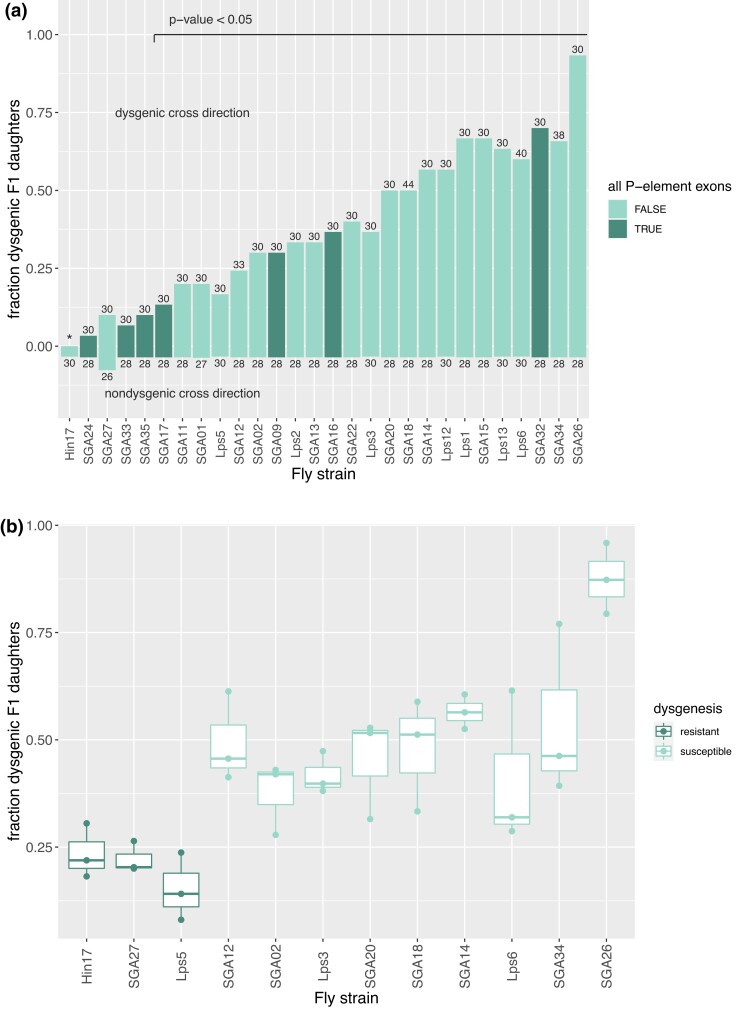
Hybrid dysgenesis in Georgia *D. simulans* lines. a) Flies from 28 lines were reciprocally crossed to a P-type tester line (Cro18), and F1 female offspring scored for dysgenesis. Data for both directions of the cross are shown above (with the Cro18 as the paternal line) and below (with Cro18 as the maternal line) the horizontal axis; the numbers above and below bars indicate sample sizes. Lines with all P-element exons in a PCR assay, and therefore with potentially active P-element, are shown in dark green. b) Flies from 12 of the lines were crossed to the P-tester line in replicate (in the dysgenic direction only), with these data used to define three lines as the most resistant (dark green) or susceptible (light green). Female F1 offspring were again dissected and the proportion showing dysgenesis scored (mean number dissected = 103, range 31–173).

For the initial screen, we reciprocally crossed each of the 28 lines to *Cro18* (a “P-type” tester line that has active *P*-elements and can induce dysgenesis. In the dysgenesis-inducing direction of the cross, there was substantial variation in the proportion of dysgenic offspring among lines, ranging from 0.20 to 0.93. Of the 28 lines, 21 showed gonadal dysgenesis, i.e. significantly more female offspring showed GD in crosses with P-type males than in the reciprocal cross, Fisher's exact test, *P* < 0.05; Supplementary Table 3; Fig. 1a.

To test if this variation had a genetic basis, we repeated the dysgenic assays for each line with replication. Because residual variation in the P-tester line (e.g. in *P*-element copy number; Hill *et al.* 2016; Serrato-Capuchina *et al.* 2020) may underlie variation in the strength of the dysgenesis phenotype, we first reduced variation within the P-tester line *via* sib-mating, creating three inbred sublines. We then used the males from these sublines to retest 12 of the Georgia lines, selected across the whole range of levels of susceptibility to HD, focusing on lines that lack complete *P*-elements. The results for this second set of crosses (Fig. 1b) were consistent with those from the initial screen above (Fig. 1a), with the proportions of dysgenic offspring for each line correlated between experiments, Pearson's correlation *r* = 0.877, *P* = 0.0004.

Consistent with nonzero broad sense heritability, we find that significant variation in dysgenesis among offspring is due to the maternal line (Fig. 1b; *n* = 36 crosses; binomial GLM with dysgenic vs nondysgenic F1s ∼ line + tester subline vs model with tester subline only, *P* < 2.2e−16). For subsequent analysis, we used the means of the repeated crosses in Fig. 1b to represent the dysgenesis phenotype for each line, as more offspring were measured for these crosses.

### No role for copy number of dysgenesis-inducing TEs

Differences in levels of dysgenesis among lines could result from differences in the number of copies of dysgenesis-inducing transposable elements between lines. For these 12 lines, however, differences in the *P*-element copy number do not appear to cause the differences in dysgenesis. In our dysgenesis assay, the lines are crossed to a tester line estimated to have ∼20 copies *via* qPCR, swamping small differences among the lines. Further, none of the 12 have full-length *P*-element in our PCR assays, and partial *P*-elements are present in both resistant and susceptible lines (Fig. 1a; Supplementary Table 2). There was no apparent relationship between the number of *P*-element exons present and the level of dysgenesis (number of amplified exons vs log-transformed resistance, Pearson's *r* = −0.334, *P* = 0.2805). Finally, we were unable to amplify *P*-elements from small numbers of flies, suggesting that the *P*-elements segregate at low copy numbers.

However, in addition to the *P*-element, the ovarian gonadal dysgenesis phenotype can be induced by the *hobo* element, another transposon of the Terminal Inverted Repeat type (Blackman *et al.* 1987; Yannopoulos *et al.* 1987). Analogous to the *P*-element, *hobo* causes gonadal dysgenesis in the offspring of crosses between *hobo* carrying males and females from “empty” lines that lack it. To explore the possible effect of *hobo* on variation in dysgenesis among lines, we estimated *hobo* copy number with qPCR.

All 12 lines and the P-tester line contain *hobo* copies, with the P-tester line containing the highest estimated number of copies (mean ± SD 23.4 ± 5.5 for *Cro18* vs 5.3 ± 2.5 for all other lines). Variation among lines in the dysgenic phenotype is associated with variation in paternal copy number (Srivastav and Kelleher 2017). Here, however, all crosses involved the same parental line, so any differences among crosses would be due to the maternal line. For maternal *hobo* copy number, we would expect to see a positive correlation between copy number and dysgenesis. All else being equal, an increase in *hobo* copies provides an increase in *hobo* transposase and in target sites for the transposase's endonuclease activity. Contrary to this expectation, we did not find a correlation between the dysgenesis phenotype and estimated *hobo* copy number (Supplementary Figure 1; *r*^2^ = 0.411, *P* = 0.272). (We were unable to perform a similar analysis for the P-element, as there were too few copies in these lines.)

These results do not preclude a role for copy number of TEs generally but do suggest that variation in copy number of TEs does not explain variation in HD here.

### Levels of maternal piRNAs do not explain variation in HD

The main mechanism protecting the germline from TE activity is thought to be the piRNA pathway, which suppresses TEs in at least three ways: through transcriptional silencing, through post-transcriptional degradation of TE mRNA [reviewed in Sato and Siomi (2018)], and, at least in the case of the *P*-element, through splicing suppression (Teixeira *et al.* 2017). If the resistance to *P*-element-induced HD seen here is due to the piRNA pathway, we expect to see more piRNAs matching the *P*-element sequence in the resistant lines than in the susceptible ones.

We, therefore, sequenced small RNAs from the ovaries of the 12 lines in Fig. 1b (with two biological replicates each) to test for differences in the abundance of piRNAs matching different TE families, including the *P*-element and *hobo*. We obtained ∼20 million reads per line (Supplementary Table 4; length distributions after trimming are shown in Supplementary Figure 2A). We mapped them to the masked *D. simulans* reference genome and a set of canonical TE reference sequences (transposon_sequence_set v9.41 downloaded from FlyBase; Supplementary Figure 2B). Approximately 40% of the reads mapped to TEs with three or fewer mismatches, with no differences between biological replicates of the same line in the proportion of reads mapping to TEs (*t* = −2.9, *P* = 1.0).

We tested for a relationship between counts of small RNA reads and resistance to HD. To do this, we mapped the small RNA reads to a database of *Drosophila* transposable elements and tested for expression differences associated with resistance to HD. Surprisingly, with the exception of one TE, there were no significant differences for small RNAs with homology to any TE, including both the *P*-element and *hobo* (linear model with resistance as a factor fit to data transformed with voom: *P*-element, *t* = −0.829; adjusted *P* = 0.714; *hobo*, *t* = 3.122, adjusted *P* = 0.156; Supplementary Table 5). The exception, a non-LTR retrotransposon (*Doc-4*), is of unknown significance but is unlikely to cause the gonadal dysgenesis seen here, which is only known from DNA transposons.

In a further attempt to recover a relationship between *P*-element cognate small RNAs and resistance to HD, we analyzed the data with two modifications: (1) we mapped only to *P*-element references (both spliced and unspliced versions), in order to capture any piRNAs primarily homologous to other elements but which might cross-react with the *P*-element transcript. (2) As piRNAs may be able to target mRNA molecules in spite of several mismatches (Brennecke *et al.* 2007), we repeated this analysis at two different levels of stringency, allowing for up to three or up to six mismatches.

**Fig. 2.**
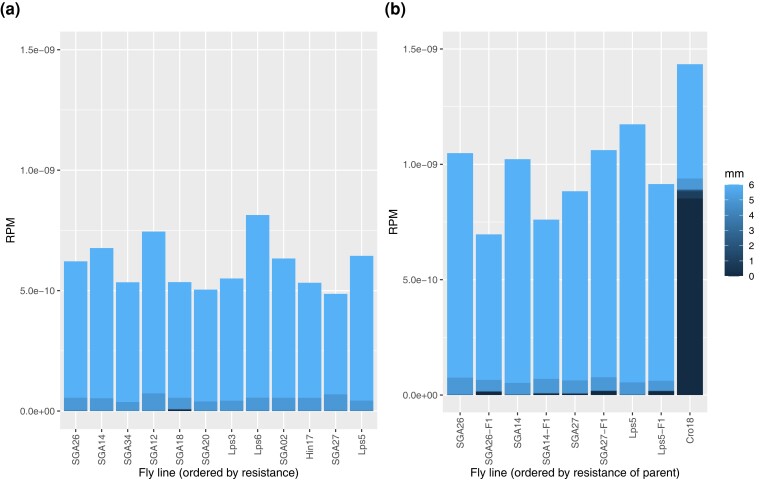
Normalized counts of small RNA reads cognate to the P-element vs resistance to hybrid dysgenesis for a) 12 lines ordered from low to high resistance, b) a subset of two susceptible and two resistant lines, and their F1 daughters when crossed to Cro18 as the paternal line. Each panel represents separate extraction and sequencing experiments. Reads-per-million (RPM) are counts are averaged across two replicates and standardized by library size; reads shorter than 22 nt were excluded (as 23 nt corresponds to the minimum length for piRNAs). The colors indicate different numbers of mismatches, with mapping up to six mismatches allowed.

With one exception (SGA18, with a moderate level of resistance), no line had any reads mapping to the *P*-element with high stringency (e.g. two or fewer mismatches; Supplementary Table 6). When filtering only for reads < 5 nt, for which matches may be spurious, there was no apparent relationship between locations of cognate *P*-element any small RNAs and resistance (Supplementary Figure 3A). When filtering for reads with lengths corresponding to piRNAs and siRNAs, there was also no apparent relationship between resistance and the abundance of these reads, regardless of mapping stringency (Fig. 2a; Supplementary Figure 3). We further compared the extreme phenotypes statistically, restricting the analysis to reads with lengths corresponding to piRNAs (23–31 nt), and allowing up to six mismatches, we compared the three most (Lps5, SGA27, and Hin17) and least (SGA26, SGA14, and SGA20) resistant lines; we saw no significant differences in coverage of the *P*-element these groups (Supplementary Table 7; *t* = 1.37, *P* = 0.655).

We reasoned that the difference between resistant and susceptible lines might instead be due to differences in the biogenesis of piRNAs cognate to the *P*-element. In particular, resistant lines may more efficiently process the piRNAs *via* the ping-pong pathway. The ping-pong pathway is the mechanism by which piRNAs are generated directly from TE mRNA—in fact, most piRNAs in the germline are generated *via* this pathway (Senti *et al.* 2015). We examined small RNA reads from 23 to 32 nt, i.e. the putative piRNAs, for a characteristic ping-pong signature (specifically, a bias for uridine at position 1 and an excess of 10 bp overlaps between sense and antisense piRNAs; Brennecke *et al.* 2007). As expected, reads mapping to TEs generally, and reads mapping to the *P*-element specifically in the Cro18 P-type line, had a strong ping-pong signal (Supplementary Figure 4). In contrast, there was no ping-pong signal for piRNAs cognate to the *P*-element in any of the 12 sequenced Georgia lines (using reads with six or fewer mismatches, as very few reads mapped with higher stringency, Supplementary Figure 5).

### Levels of piRNAs cognate to *P*-element in F1 daughters do not differ between resistant and susceptible lines

It may be that the resistant lines are indeed more efficient at generating piRNAs *via* the ping-pong pathway from TE mRNA, but this can only occur in the presence of substantial levels of *P*-element mRNA. We therefore measured piRNA levels in F1 daughters derived from crosses of four of these lines with males from the P-type tester line (Cro18), which are expected to express *P*-element mRNA. Specifically, we crossed females from the two most (Lps5 and SGA27) and least (SGA26 and SGA14) resistant lines to Cro18, and sequenced small RNAs from the ovaries of the F1 daughters and of all five parental lines. As this is the dysgenic direction of the cross, we performed these crosses at 25°C to avoid substantial gonadal atrophy and simultaneously re-sequenced ovarian small RNAs from the five parental lines.

The P-type tester line had significantly higher expression of piRNAs with sequence matches to the *P*-element compared with the other lines (Fig. 2b; *t* = 7.00, *P* = 0.0007). The F1 daughters also showed elevated expression of *P*-element piRNAs relative to their female parents (*t* = 3.5, *P* = 0.022), but there were no differences in *P*-element-derived piRNA levels between F1 daughters from the two low and two high-resistance lines (*t* = −3.7, *P* = 0.88), suggesting that increased production of piRNAs *via* ping-pong from paternal mRNA is not a major contributor to variation in resistance to HD.

### Variation in splicing of the *P*-element transcript

*P*-element activity is regulated *via* splicing suppression in the soma, with retention of the third intron (IVS3) leading to a premature stop codon, with the translation of the unspliced RNA yielding a repressor of *P*-element transposition (Black *et al.* 1987). In the germline of *D. melanogaster*, *P*-element mRNA is efficiently spliced only in the dysgenic direction of the cross, ultimately resulting in the expression of the dysgenesis phenotype. Splicing of *P*-element mRNA in the germline is suppressed via piRNA pathway (Teixeira *et al.* 2017; Moon *et al.* 2018).

We tested whether differences in splicing regulation among lines underlie differences in the *P*-element dysgenic phenotype. To this end, we crossed 10 of our lines to the P-tester line in both directions and at two different temperatures (25°C and 29°C), and measured *P*-element expression and splicing efficiency using qPCR. We measure the abundance of spliced transcripts using a forward primer in exon two paired with a reverse primer bridging an intron (IVS3), which matches only transcripts where IVS3 is removed. We measured total *P*-element expression using the same forward primer and a reverse primer within the same exon. We estimated splicing efficiency as the ratio of spliced to total expression and analyzed both splicing efficiency and the raw expression results with a linear model (response variable either spliced expression or splicing efficiency ∼ cross-direction (dysgenic/reciprocal) + temperature + maternal line; Supplementary Table 8).

**Fig. 3.**
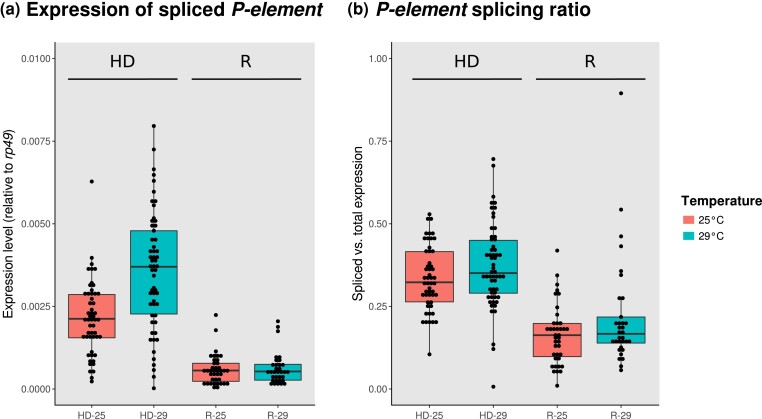
*P*-element expression and splicing rates. a) Expression rate of spliced *P*-element measured using qPCR in F1 ovaries of dysgenic (HD) and reciprocal (R) crosses, at 25°C (red; left box of each pair) and 29°C (green; right box for each pair). b) Splicing ratio of the *P*-element in the same conditions (spliced vs total expression).

Consistent with the dysgenic phenotype, offspring of dysgenic crosses have both significantly higher expression of spliced *P*-elements and significantly higher splicing efficiency than offspring from the reciprocal cross, particularly at 29°C (Fig. 3; Supplementary Figure 6). Importantly, maternal lines differ in *P*-element expression and splicing efficiency, raising the possibility that these differences underlie variation among the lines in resistance. Indeed, under conditions when dysgenesis is induced, there is a significant association between resistance to *P*-induced dysgenesis and splicing efficiency (ANOVA; *F*_1, 98_ = 5.950, *P* = 0.017), showing a potential role for regulation of splicing in the suppression of HD. Nevertheless, there is still substantial remaining variation in resistance to *P*-element dysgenesis, as illustrated by the significant effect of line in the analysis after accounting for resistance (ANOVA; *F*_8, 98_ = 5.300, *P* = 0.016), and essentially equal splicing between lines with the highest and lowest resistance (Supplementary Figure 6).

### Resistance to HD is recessive

**Fig. 4.**
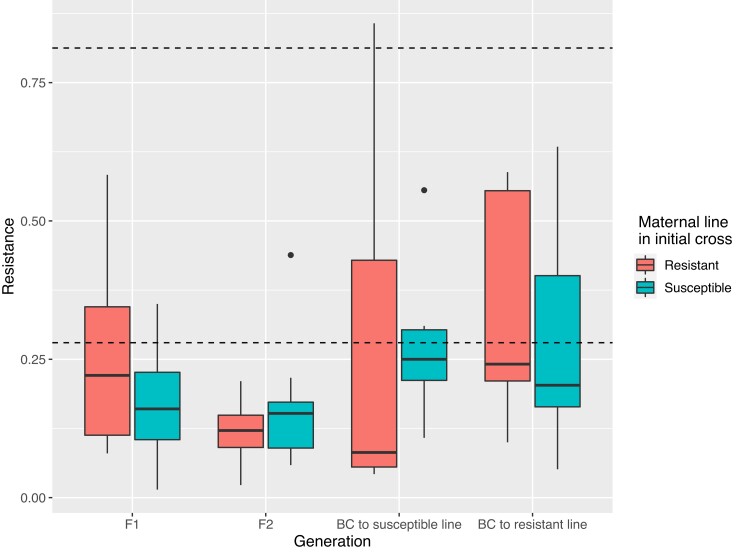
Genetics of resistance to hybrid dysgenesis. Boxplots show hybrid dysgenesis phenotypes of the offspring of reciprocal crosses between two resistant and two susceptible lines, with data from different crosses pooled by generation. Horizontal lines show the mean resistance of the resistant (top line) or susceptible (bottom line) parental lines. The initial cross was performed in both directions; “RS’ indicates that the maternal line was resistant, “SR” that it was susceptible.

We examined the inheritance pattern of resistance. We reciprocally crossed the two most (Lps5 and SGA27) and least tolerant lines (SGA26 and SGA14; eight total crosses), and measured the resistance of the F1 daughters to HD. (Note that the F1 resistance phenotype is measured by scoring F2 daughters for dysgenesis). In general, the phenotype of the F1 was similar to that of the most susceptible parent, showing that resistance to HD is at least partially recessive (Fig. 4; Supplementary Figure 7; Supplementary Table 9). This is consistent with the lack of a strong piRNA-related effect on resistance, as a piRNA-driven resistance would, at least naïvely, be expected to be dominant.

If resistance is piRNA-mediated, we also might expect a “grandmother effect”, wherein the phenotype of the grandmother affects the phenotype of subsequent generations (de Vanssay *et al.* 2012). We, therefore, performed an additional set of crosses to obtain backcross and F2 offspring (Supplementary Table 10). In these crosses, F1s from reciprocal crosses involving one resistant line (Lps5) show patterns of resistance consistent with a grandmother effect (Supplementary Table 10; Supplementary Figure 7B). That said, in *D. melanogaster*, the grandmother effect increases in subsequent generations (de Vanssay *et al.* 2012). Here, we see no effect of the initial direction of the cross consistent with a grandmother effect after the F1 (Fig. 4; Supplementary Figure 7). Rather, in a comprehensive analysis of the data, we find that the major factor explaining resistance was the expected contribution of nuclear genes from the resistant line, with no significant effect of cross-direction or line identity (Supplementary Table 10; binomial GLM, *z* = 1.021, *P* = 0.009).

## Discussion

We find that there is substantial genetic variation in resistance to *P*-element-induced ovarian dysgenesis in *D. simulans* lines collected during the early phase of the *P*-element invasion. Further, we find that this variation does not appear to be a simple function of the amount of *P*-element cognate small RNAs in ovaries of resistant lines. Given the extensive evidence demonstrating that piRNAs are the most important defense against TEs in the germline, our results are unexpected. In addition to many studies characterizing the suppressive effect of piRNAs on transposable elements generally (reviewed in Ozata et al. (2019)], variation in levels of *P*-element cognate piRNAs has been associated with the strength of HD suppression in *D. melanogaster* (Wakisaka *et al.* 2018). In *D. simulans*, too, the evolution of *P*-element suppression in laboratory populations co-occurred with the evolution of piRNAs acting against the *P*-element (Kofler *et al.* 2018). Similarly, we find abundant *P*-element piRNA expression in the P-type Cro18 line, which does not show dysgenesis in spite of harboring multiple copies of the *P*-element.

Our findings do not preclude a role for piRNAs or other small RNAs in explaining levels of resistance among the studied lines. Here, we measured total expression in whole ovaries, but variation in the developmental timing of this expression, for example, could lead to variation in the effectiveness of *P*-element silencing. Alternatively, the lines could vary in downstream components of silencing. For instance, piRNAs regulate TEs in at least three ways: by degrading TE transcripts *via* cleavage by Argonaute proteins in the cytoplasm, as is typical for small RNAs, by inducing chromatin state changes that transcriptionally silence TEs in the nucleus, and by splicing suppression, at least in the case of the *P*-element (Brennecke *et al.* 2007; Klenov *et al.* 2007, 2011; Senti and Brennecke 2010; Wang and Elgin 2011; Le Thomas *et al.* 2013; Teixeira *et al.* 2017). Variation in any one of these downstream factors [reviewed in Lee and Langley (2012)] might lead to variation in epigenetic suppression of TEs, as has been seen in comparisons between species (Lee and Karpen 2017). Host factors that interact directly with the *P*-element (e.g. *P-splice inhibitor* (Adams *et al.* 1997) are also potential sources of variation in the level of P tolerance). The fact that the splicing efficiency of the *P*-element appears to be weakly correlated with resistance particularly implicates that aspect of piRNA-mediated *P*-element silencing.

Alternatively, variation in resistance could be independent of small RNAs and mediated by factors that do not interact with or target the *P*-element transcript directly but regulate the response to the damage caused by *P*-element activity. HD is thought to be a consequence of apoptosis of developing germline cells, triggered by double-stranded DNA breaks, inherent to *P*-element transposition (Dorogova *et al.* 2017). Thus, resistance to dysgenesis may be affected by variations in the efficiency of DNA repair among the lines, as in *D. melanogaster* (Lama *et al.* 2021). Similarly, factors involved in apoptosis and germline stem-cell maintenance explain some non-piRNA dysgenesis tolerance in *D. melanogaster* (Tasnim and Kelleher 2017; Kelleher *et al.* 2018), though note that these act zygotically, rather than maternally as seen here.

This and other work (e.g. Tasnim and Kelleher 2017; Kelleher *et al.* 2018) demonstrate that the full complement of molecular mechanisms that affect TE defense is still being identified, particularly those independent of a fully functioning piRNA-based defense. Defenses not relying on piRNAs may be especially important early in a TE invasion. Once a TE is established in a genome, piRNAs may act to suppress specific TEs and serve as a memory of previous TE activity, analogous to the adaptive immune response (Siomi *et al.* 2011). But, in the early stages of invasion, mechanisms not specific to a given TE act to mask the negative consequences of transposition and to ensure the survival of the cells, analogous to a nonspecific “innate” immune response.

## Supplementary Material

jkac324_Supplementary_Data

## Data Availability

Small RNA data are deposited at NCBI (http://www.ncbi.nlm.nih.gov/) under BioProject ID PRJNA553233. Supplemental material is available at G3 online.

## Notes

*Communicating editor: J. Blumenstiel*
