# Supplementary material for: Levels of P-element-induced hybrid dysgenesis in Drosophila simulans are uncorrelated with levels of P-element piRNAs:
Source: G3 (Bethesda). 2022 Dec 8;13(2):jkac324. doi: 10.1093/g3journal/jkac324 (PMC9911080; doi:10.1093/g3journal/jkac324)
Supplement: jkac324_Supplementary_Data [file jkac324_supplementary_data.zip › Supplemental_Figures_G3-2022-403869.docx]

**Figure S1.** *Hobo* copy number as estimated by qPCR in the maternal strain crossed vs. resistance to hybrid dysgenesis when crossed to *Cro18*. Resistance data are means across replicates shown in Figure 1B.

**A**

**B**

**Figure S2.** A) Lengths of unmapped small RNA reads from each of the 12 lines, after adapter removal but before any further processing. B) Lengths of small RNA reads mapping to the transposable element library with three or fewer mismatches from each of the 12 lines.

A) Read length ≥ 5 nts

B) Read length 21-22 nts

C) Read length ≥ 21nt

**Figure S3. Normalized counts of smallRNA reads cognate to the P-element vs. resistance to hybrid dysgenesis.** Left panels show data for 12 strains ordered by resistance to P-element induced hybrid dysgenesis. Right panels show data for a subset of two susceptible and two resistant strains and their F1 daughters (when crossed to Cro18 as the paternal line). Left and right panels represent separate extraction and sequencing experiements.

Reads per million (RPM) are counts are averaged across two replicates and standardized by library size. Reads lengths included in each plot are A) all reads greater than 5nt, B) reads corresponding to the length of siRNAs, 21-22nt, and C) reads corresponding to the length of siRNAs and piRNAs together, ≥ 21 nt. The colors indicate different numbers of mismatches, up to six mismatches (abbreviated ‘mm’).

A

B

C

D

**Figure S4.** Locations of small RNAs mapping to the P-element in *D. simulans* strains that vary in their resistance to hybrid dysgenesis. Coverage depth for reads longer than 21 nt and mapping with 3 or fewer mismatches is shown; raw, unnormalized counts are shown for clarity. (Library sizes are given in Table S3.) The top two panels show data from 12 strains, ordered by increasing resistance to hybrid dysgenesis, with raw coverage depth for (A) the P-element reference and (B) the P-element mRNA. For contrast, the same analysis was performed for the P-tester strain, Cro-18, which both induces and is resistant to the hybrid dysgenesis phenotype, with raw coverage of the (C) the P-element reference and (D) P-element mRNA shown.


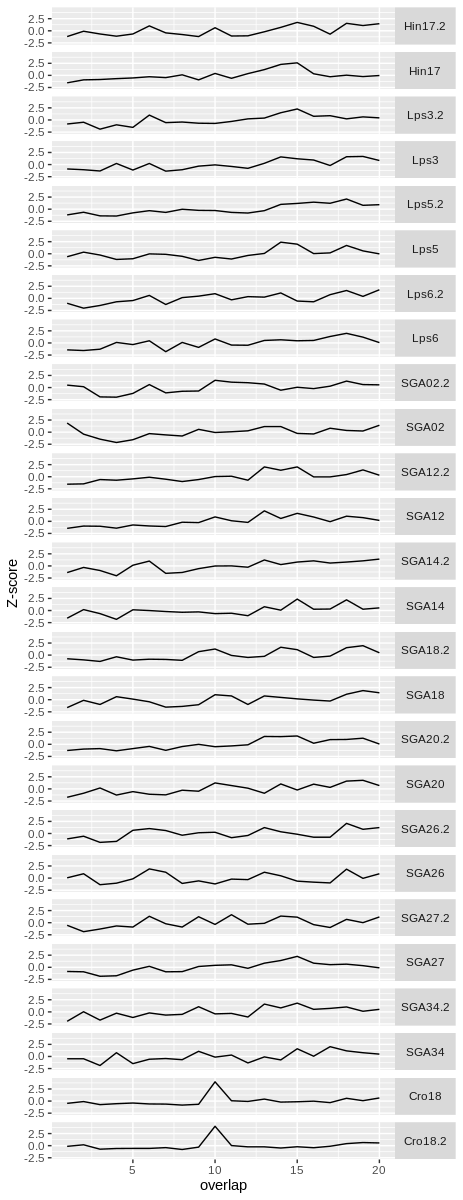


**Figure S5. Ping pong signature of P-element-specific piRNAs.** Z-score of sense-antisense read pairs mapped to P-element sequences overlapping by 1 to 20 nucleotides; 10nt overlap corresponding to ping pong signal. For Cro18, mapping with up to 3 mismatches shows a clear ping pong signal (see peak at 10nt overlap in bottom two rows). For other lines, there is no clear ping-pong signal with either 3 mismatches (where there were no overlapping read pairs), or 6 mismatches (shown above; note no peak at 10nts). Cro18 also failed to show a clear ping-pong signal with 6 mismatch read mapping due to too many non-specific alignments.

**
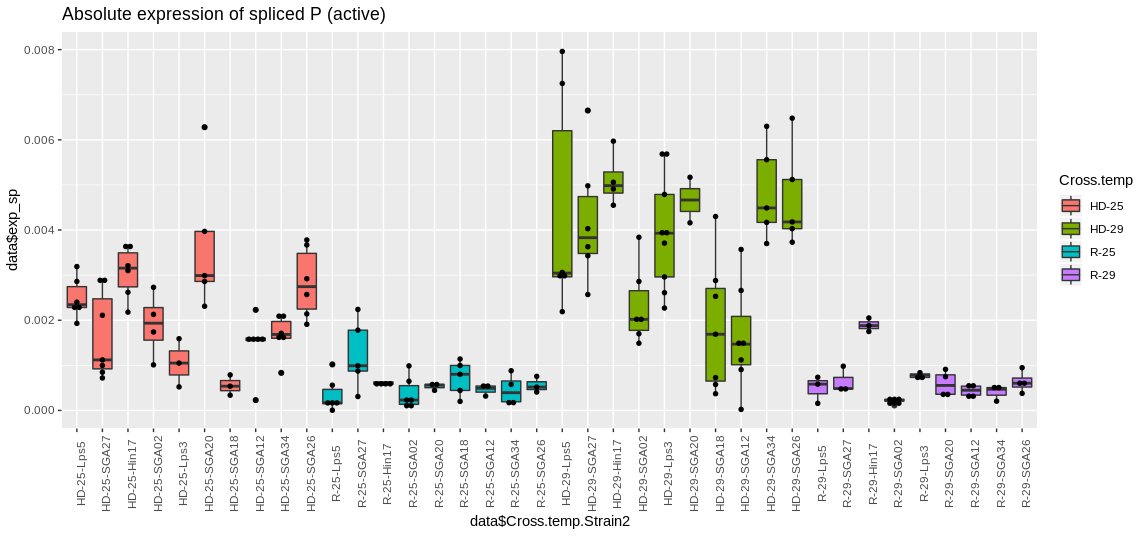
**

**A**

**B**

**
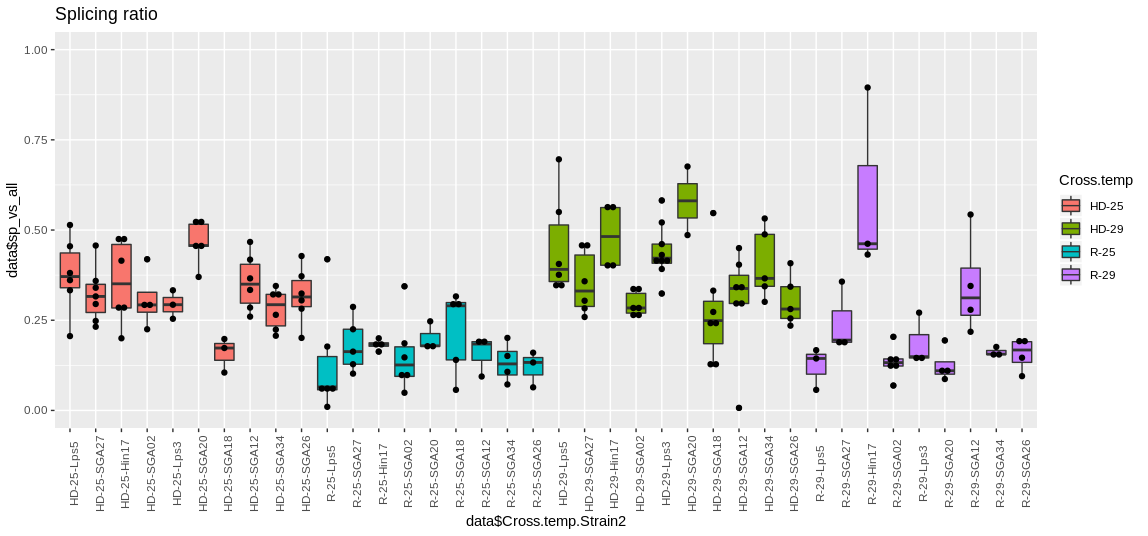
**

**Figure S6.** P-element expression and splicing efficiency for all crosses, with results from each cross plotted separately (as points, and summarised in boxplots). Strains are ordered from left to right as most to least resistant. Each of nine strains was crossed to the P-type tester line in the dysgenic (HD) and Reciprocal (R) direction, and RNA extracted from ovaries of F1 daughters. qPCR was performed using primers that amplify either exon 2 (amplifying both spliced and unspliced transcripts) a region that includes part of exon 2 and of the intron between exons 2 and 3 (amplifying only unspliced transcripts). The amount of spliced transcript and splicing efficiency was estimated from the unspliced vs. total transcript levels. Transcripts from reference gene (rp49) were amplified simultaneously **A)** Expression of spliced P-element relative to the reference and **B)** splicing efficiency at 25 ºC and 29 ºC in dysgenic (HD) and reciprocal (R) crosses. Note that dysgenesis is not normally expressed at 25ºC.


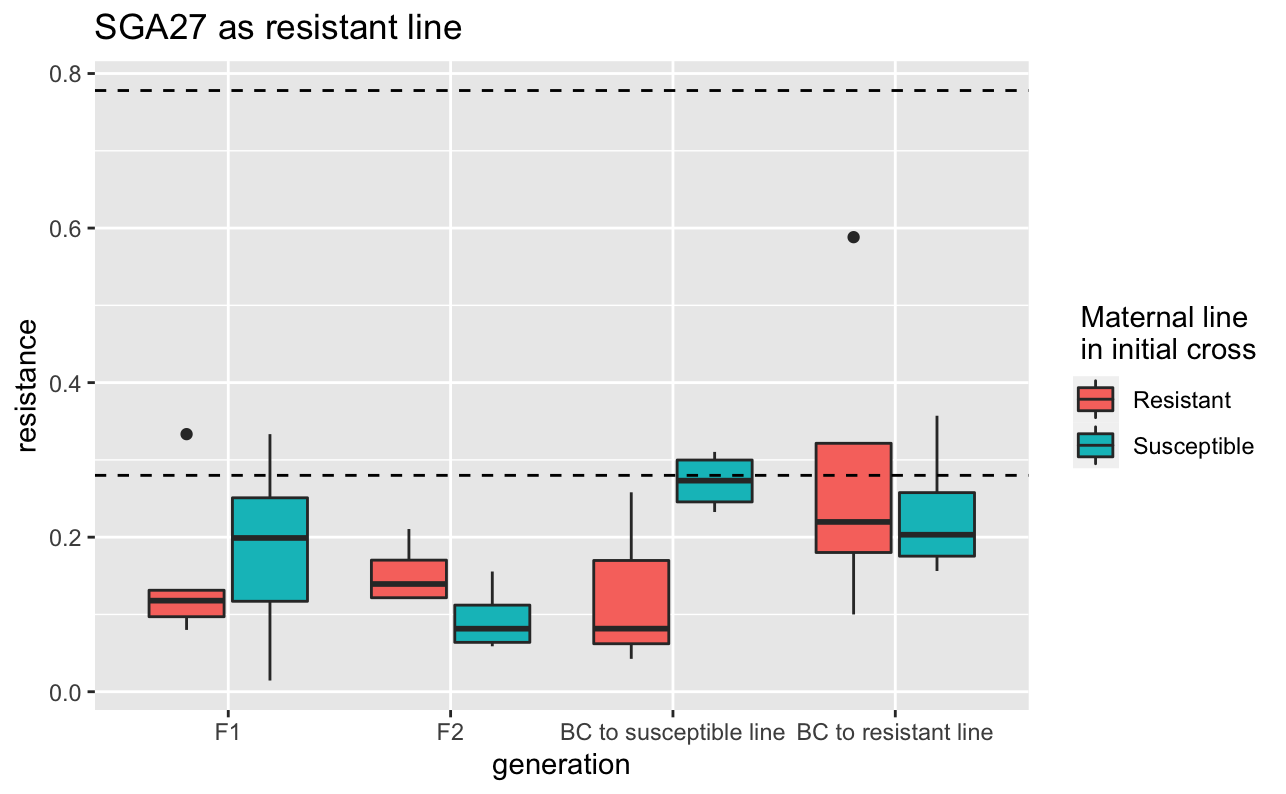

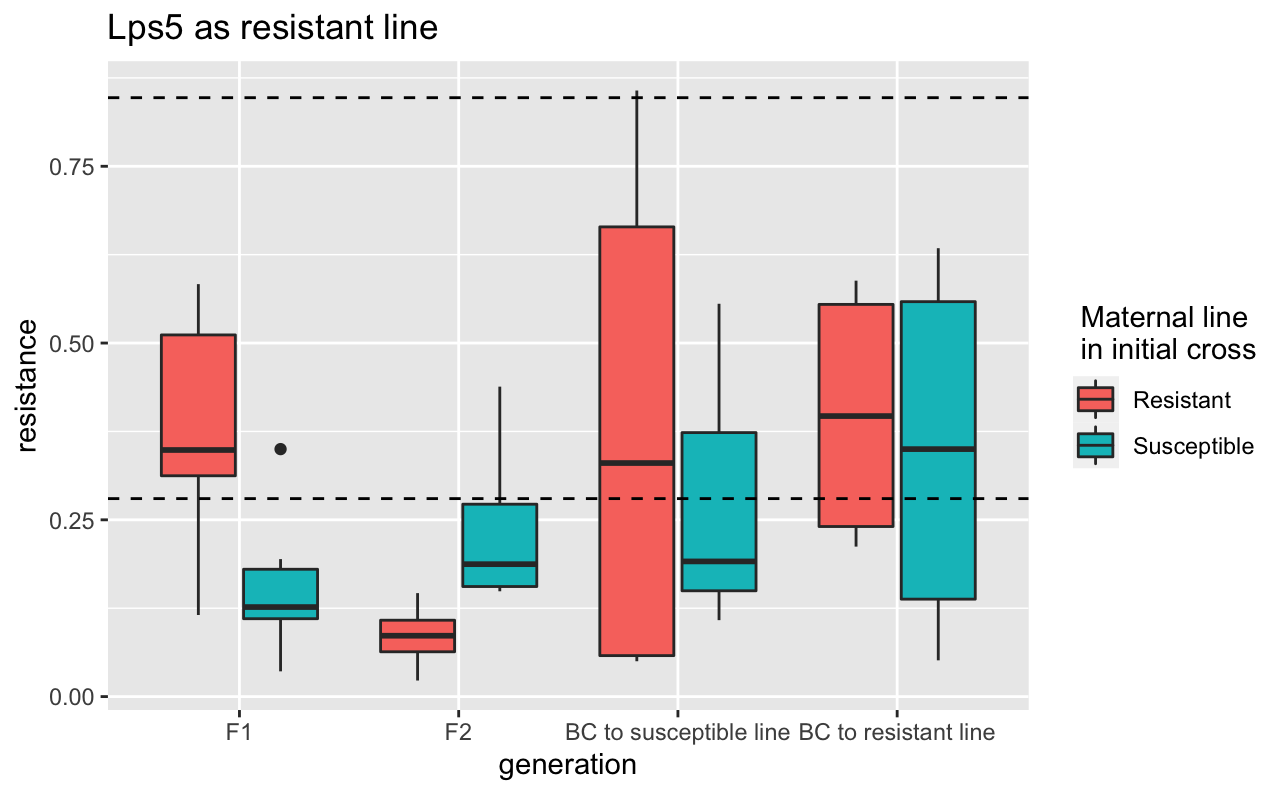


**Figure S7. Genetics of resistance to hybrid dysgenesis.** Boxplots show hybrid dygenesis phenotypes of the offspring of reciprocal crosses between resistant lines SGA27 (top) and Lps5 (bottom) and two susceptible lines, with data from different crosses pooled by generation. Horizontal lines show the resistance of the resistant (top line) or mean of the susceptible (bottom line) parental lines. The initial cross was performed in both directions; ‘RS’ indicates that the maternal line was resistant, ‘SR’ that it was susceptible.
